# Progress in Surgery

**Published:** 1900-02-10

**Authors:** 


### Progress in Surgery.

### SURGERY OF THE LIVER AND GALL-BLADDER.

**Treatment of Ascites of Cirrhosis by the Artificial Production of Peritoneal Adhesions.**—Rolleston and Turner[1] give a brief account of two cases treated by this method, and discuss certain points in the technique of the operation and the *rationale* of the treatment. Of the two cases referred to one of the patients improved, lost the ascites almost entirely, and felt much better in health, but was left with an enlarged spleen, so that he cannot as yet be regarded as cured. In the other case the ascites recurred again and again; and though the patient's general state was much better than before the operation, when it appeared that life could not be much prolonged, the result can at best be regarded as only a slight amelioration.

The authors regard it as important that the operation should not be postponed until the patient is too debilitated to withstand it, for patients with cirrhosis are prone to secondary infections, especially to peritonitis. When a course of iodide of potassium has not benefited a case of ascites which is thought to be due to either syphilis or cirrhosis, medical treatment should be suspended and operative measures employed.

An oblique incision skirting the costal margin gives better access to the upper liver surface on both sides of the suspensory ligament, and enables the omentum to be stitched on that surface between it and the diaphragm better than does an incision in the senilunar line. Suprapubic drainage is necessary in cases where there is a large amount of fluid present, and where there has been a rapid re-accumulation after previous tappings. The sitting posture is more advantageous than the recumbent one, as it tends to allow of the more satisfactory formation of adhesions between the omentum and liver, and the abdominal parietes.

The improvement, both in the general health and in the local condition—viz., the disappearances of ascites—has been supposed to depend simply on the collateral circulation relieving the pressure in the portal vein. The authors think there are two possible ways in which the development of a collateral circulation in adhesions around the liver may be beneficial to the economy. (1) By somewhat diminishing the flow of blood through the liver, it may enable that organ to deal more satisfactorily with the blood passing through it, and so reduce the important factor in inducing ascites. (2) That the increased vascular supply to the surface of the liver may, by improving the nutrition of the hepatic cells, enable them to undergo compensatory hyperplasia. The compensatory hypertrophy will enable the organ to perform more efficiently its important antitoxic functions, and so lead to a latency of the symptoms. If the last hypothesis be true, it is evidently of importance that any operation for the formation of vascular adhesions around the liver should be undertaken before the liver tissue is so disorganised that compensatory hyperplasia is impossible.

**Resection of the Liver** for hepatic tumour has been successfully performed three times by Keen.[2] In the last case reported, the left lobe of the liver was affected with carcinoma, and the entire lobe, and with it the whole of the disease, was severed from the rest of the liver by means of the Paquelin cautery. The hæmorrhage was not severe, except when the cautery divided some of the larger veins, and these were secured by a catgut ligature. A part of the raw surface left on removal of the left lobe was closed by folding the edge of the liver upon itself, like an amputation flap, and the remaining part was covered by a packing of iodoform gauze. The author thinks that an elastic ligature is advisable only in exceptional cases.

Thompson[3] also reports a case of removal of a large growth occupying contiguous parts of the right and left lobes, and covered by the suspensory ligament. The growth was cut away with curved scissors, after provisional control of the hæmorrhage, by long needles and an elastic ligature, and the raw surface was sutured to the margins of the abdominal wound. The growth removed was found to be gummatous. The patient recovered.

**Hydatid of the Liver.**—Jones[4] reports a case of this disease in a girl aged eight years. Operation was at first declined, and the cyst suppurated and ultimately burst into a bronchial tube. It was inferred that an œdema of the skin covering the chest indicated that the ulcers had burst into the lung at no great depth from the surface, or else that it must have burst primarily into the pleural cavity, and secondarily into the lung. It was, therefore, concluded that less immediate danger would ensue from an incision into the chest than from an incision through the abdominal wall, and this was therefore made immediately below and internal to the nipple. Sinus forceps were introduced through the pleura into the lung tissue, and fœtid pus then escaped. The wound was enlarged and a drainage tube inserted. Some hours later the whole of the cyst wall was removed through this incision. The patient recovered.

**Cholelithians.**—Ferguson[5] claims that biliary calculi are foreign bodies, and that they should therefore be treated surgically. In first attacks of hepatic colic tentative treatment is allowable, but the patient should be warned that the attacks will in all probability recur and a surgical operation may be necessary to save his life. But in acute attacks, attended with jaundice, and lasting longer than from ten days to two weeks, and in which there are no signs of improvement, operation is indicated. In all acute cases, unattended with jaundice, lasting for a week or more without improvement, operation is unmistakably indicated. In all chronic cases, namely, cases in which there has been a recurrence from time to time of hepatic colic, whether there is or is not a coincident jaundice, operation is indicated. Richardson[6] also thinks that operation is called for whenever gall-stones are believed to be present. After removal of the calculi he prefers to suture the gall-bladder to the abdominal wound, as leaving the gall-bladder closed in the abdominal cavity has been, in his experience, a dangerous procedure. Weir[7] agrees that after incising the gall-bladder and removing the stones it is well to stitch the bladder to the external wound and leave the latter open. Cases of obstruction of the common duct are of two classes, those representing few adhesions, and those having many adhesions. In the former class, with the finger in the foramen of Winslow the surgeon can command the gall passages fairly satisfactorily.

Utilising some experiments by Jones on dogs for the cure of gall-bladder fistula, McBurney[8] has tried a similar method on the human being with success. The method consists in inverting the orifice made by the surgeon's knife, so that on closure of the wound this orifice was inverted and the peritoneal coats are in contact. Through this inverted orifice the drainage tube is inserted for the short period required. When treated in this way the fistula would close within 48 hours after the removal of the tube.

[1] Lancet, Dec. 16, 1899, p. 1660. [2] Brit. Med. Jour., Nov. 25, 1899, Epitome No. 407; and Annals of Surgery, Sept., 1899. [3] Ibid. [4] Lancet, Nov. 25, 1899, p. 1435. [5] New York Med. Rec., Oct. 7, 1899, p. 508. [6] New York Med. Rec., Nov. 25, 1899, p. 801. [7] Ibid., p. 801. [8] Ibid., p. 802.

